# Phenotypic and genotypic investigation of metallo-β-lactamases in *Pseudomonas aeruginosa* clinical isolates in Bushehr, Iran

**DOI:** 10.22038/IJBMS.2022.64359.14154

**Published:** 2022-10

**Authors:** Mahboubeh Seyedi, Forough Yousefi, Behrouz Naeimi, Saeed Tajbakhsh

**Affiliations:** 1 Department of Microbiology and Parasitology, School of Medicine, Bushehr University of Medical Sciences, Bushehr, Iran; 2 Student Research Committee, Bushehr University of Medical Sciences, Bushehr, Iran; 3 The Persian Gulf Tropical Medicine Research Center, The Persian Gulf Biomedical Sciences Research Institute, Bushehr University of Medical Sciences, Bushehr, Iran

**Keywords:** blaI_MP_, bla_NDM_, bla_VIM_, Metallo-β-lactamase, MBL, Pseudomonas aeruginosa

## Abstract

**Objective(s):**

Production of metallo-β-lactamases (MBLs) is an important mechanism of resistance to carbapenems. This study aimed to detect the MBL-producing *Pseudomonas aeruginosa* clinical isolates and to investigate the presence of *bla*_VIM_, *bla*_IMP_, *bla*_SPM_, *bla*_NDM_, *bla*_GIM_, *bla*_AIM_, and *bla*_SIM_ genes in these isolates in Bushehr, Iran.

**Materials and Methods:**

A total of 169 *P. aeruginosa* clinical isolates were collected from three hospitals in Bushehr. The modified carbapenem inactivation method (mCIM) was used for the phenotypic detection of carbapenemase production. A combination disk test (CDT) was performed for the phenotypic detection of MBL production. To investigate the presence of *bla*_VIM_, *bla*_IMP_, *bla*_SPM_, *bla*_NDM_, *bla*_GIM_, *bla*_AIM_, and *bla*_SIM_ genes, PCR and sequencing was carried out.

**Results:**

Based on the results of mCIM, 40 (23.7%) of 169 isolates were carbapenemase producers. CDT revealed that 26 (15.4%) isolates were MBL producers. *bla*_IMP_, *bla*_NDM_, and *bla*_VIM_ genes were found in 18 (69.2%), 8 (30.8%), and 1 (3.8%) of the MBL-producing isolates, respectively. Coexistence of *bla*_IMP_ and *bla*_NDM_ was observed in 2 (7.7%) MBL-producing isolates. Among all 169 *P. aeruginosa* isolates, 23 (13.6%) harbored *bla*_NDM_, 18 (10.6%) carried *bla*_IMP_, and 1 (0.6%) carried the *bla*_VIM_ gene. *bla*_SPM_, *bla*_GIM_, *bla*_AIM_, and *bla*_SIM_ were not found in the present study.

**Conclusion:**

*bla*_NDM_, *bla*_IMP_, and *bla*_VIM_ genes were detected in this study, which could be a warning sign about the prevalence of these genes among *P. aeruginosa* clinical isolates in our region. Proper monitoring and detection of MBL-producing isolates are essential steps to prevent the spread of these isolates.

## Introduction

*Pseudomonas* *aeruginosa* is an opportunistic pathogen that is considered an important cause of nosocomial infections (1). This microorganism is responsible for a wide spectrum of infections including pneumonia, bacteremia, urinary tract infection, and skin infection. *P*. *aeruginosa* is intrinsically resistant to a number of antibiotics and is capable of developing resistance to almost any available antimicrobial compound (2). Because of its intrinsic and acquired drug resistance, only limited classes of antibiotics are effective for the treatment of *P*. *aeruginosa* infections. Among these antibiotics, carbapenems have been considered the most potent β-lactams. Carbapenems are amongst the antimicrobial agents which are used for treatment of multidrug-resistant *P*. *aeruginosa*. Therefore, resistance to carbapenems is challenging in clinical settings (3).

Carbapenem resistance in *P*. *aeruginosa* is often due to impermeability which arises via the loss or alteration of the OprD porin, overexpression of efflux pumps (2, 4, 5, 6), or acquisition of foreign genes encoding Ambler class A, class B, or class D β-lactamases able to hydrolyze carbapenems at various degrees (3, 6). Metallo-β-lactamases (MBLs) belong to Ambler class B, are the most frequent carbapenemases involved in *P*. *aeruginosa* carbapenem resistance (2). In recent years, *P*. *aeruginosa* isolates producing MBLs of different types such as GIM, IMP, NDM, SPM, and VIM have been reported with an increasing frequency worldwide (7). MBLs, which use zinc for hydrolysis of the β-lactam ring, are inhibited by metal chelators, such as ethylenediaminetetraacetic acid (EDTA) and thiol-based compounds (3, 5), but are not inhibited by clavulanic acid, tazobactam, and sulbactam (3, 8). Metallo-β-lactamases confer resistance to all β-lactam antibiotics except monobactams (3).

The MBL genes are located on specific genetic elements including integrons, transposons, plasmids, or on the chromosome. These genetic elements are transferable to other Gram-negative organisms (3, 5), increasing the antimicrobial resistance rate and complicating the therapy of infected patients (3). Infections caused by MBL-producing isolates are associated with high morbidity and mortality rate, particularly in hospitalized and immunocompromised patients (9). The prevalence of MBL-producing *P*. *aeruginosa* and MBL genes may vary in different geographical areas. Therefore, it is necessary to identify and control these organisms in each geographical area (8). This study aimed to detect the MBL-producing *P*.* aeruginosa* clinical isolates and to investigate the presence of *bla*_VIM_, *bla*_IMP_, *bla*_SPM_, *bla*_NDM_, *bla*_GIM_, *bla*_AIM_, and *bla*_SIM_ genes in these isolates in the city of Bushehr, southwest of Iran.

## Materials and Methods

***P. aeruginosa clinical isolates***

This study was approved by the Ethical Committee of Bushehr University of Medical Sciences, Iran, with reference number IR.BPUMS.REC.1398.082. A total of 169 *P*.* aeruginosa* clinical isolates recovered from clinical specimens including urine, endotracheal tube (ETT) secretions, blood, wound, throat, eye, ureter secretions, ear, and catheter (catheter culture to aid in the diagnosis of catheter-related infection), were collected from three hospitals in Bushehr, Iran, between May 2017 and August 2019. Identification of the isolates was carried out by conventional bacteriological methods and confirmed by PCR using specific primers to target 16S rDNA of *P*. *aeruginosa* (10) (Table 1).

***Antimicrobial susceptibility testing***

Antimicrobial susceptibility testing for carbapenems including imipenem, meropenem, and doripenem was performed by the disk diffusion method and interpreted according to the Clinical and Laboratory Standards Institute (CLSI 2018) (11). Imipenem disk (10 µg), meropenem disk (10 µg), and doripenem disk (10 µg) (Mast Ltd, Merseyside, UK) were used. *Pseudomonas aeruginosa* ATCC 27853 and *Escherichia coli *ATCC 25922 were used for quality control. The minimum inhibitory concentration (MIC) of imipenem was determined for the isolates that were resistant to this antibiotic in the disk diffusion method. MIC Test Strip (Liofilchem, Italy) was used to determine the MIC of imipenem.

All tested isolates that were not susceptible to one or more carbapenems, were chosen for the phenotypic detection of carbapenemase production (11). 

***Modified carbapenem inactivation method (mCIM)***

Isolates that were intermediate or resistant to one or more carbapenems, were selected for phenotypic detection of carbapenemase production. mCIM can detect serine carbapenemases and metallo-β-lactamases (11). The mCIM test was performed for each isolate according to the CLSI 2018 procedure as follows: briefly, a 10-µl loopful of bacteria from an overnight blood agar plate was emulsified in 2 ml tryptic soy broth (TSB). A 10-µg meropenem disk was added to the tube so that the entire disk was immersed in the suspension. The tube was incubated at 35 ^°^C for 4 hr±15 min. Just before completion of the TSB-meropenem disk suspension incubation, a 0.5 McFarland suspension of *E*. *coli* ATCC 25922 (that is a meropenem-susceptible strain) was prepared and inoculated on a Mueller Hinton agar plate as for the routine disk diffusion procedure. Afterward, the meropenem disk was removed from TSB-meropenem disk suspension and placed on the Mueller Hinton agar plate previously inoculated with *E*. *coli *ATCC 25922. The Mueller Hinton agar plate was then incubated at 35 ^°^C for 18-24 hr. Following incubation, the zone of inhibition was measured and the results were interpreted according to CLSI. A zone diameter of 6-15 mm or the presence of pinpoint colonies within a 16-18 mm zone was considered carbapenemase positive (mCIM positive). A zone diameter of ≥19 mm was considered carbapenemase negative (mCIM negative) (11). A carbapenemase-producing strain of *P*. *aeruginosa* was used as positive control, and *P*. *aeruginosa* ATCC 27853 was used as negative control.

***Combination disk test (CDT)***

*P*. *aeruginosa* isolates that were intermediate or resistant to one or more carbapenems (5, 11), were also tested by CDT for the phenotypic detection of MBL production. CDT with imipenem (CDT-IMI) was carried out for each isolate as follows: a bacterial suspension with turbidity equal to 0.5 McFarland was prepared and inoculated on Mueller Hinton agar II (Biolife, Milano, Italy) plates as for the disk diffusion procedure. Two imipenem (10 µg) disks were placed on the Mueller Hinton agar II plate, then, 5 µl of 0.5 M EDTA (930 µg EDTA) was added to one of the imipenem disks. Since EDTA has some bactericidal activity, a blank disk without antibiotics was also inoculated with 5 µl of 0.5 M EDTA (5). The plates were incubated overnight at 35 ^°^C. After incubation, an increase of ≥7 mm in zone diameter around imipenem plus EDTA disk in comparison with imipenem disk demonstrated the production of MBL by the bacterium (1, 5, 12). Furthermore, CDT-IMI with 750 µg EDTA was also performed and interpreted similarly (13).

Moreover, at the same time, CDT with 10 µg meropenem disks (CDT-MEM) was done by using 930 µg EDTA and 750 µg EDTA. The procedure and interpretation of CDT MEM results were similar to that described above (5).

A metallo-β-lactamase-producing strain of *P*. *aeruginosa* was used as positive control, whereas *P*. *aeruginosa* ATCC 27853 was applied as negative control (5).

***Detection of MBL genes by PCR assay and sequencing***

The total DNA from *P*. *aeruginosa* isolates was extracted using an extraction kit (GeneAll Biotechnology Co., Ltd, Seoul, South Korea) according to the manufacturer’s directions. Specific primers for the detection of MBL genes (*bla*_VIM_, *bla*_IMP_, *bla*_SPM_, *bla*_NDM_, *bla*_GIM_, *bla*_AIM_, and *bla*_SIM_) were used (14) (Table 1). Amplification of targeted DNA was carried out in 25 µl reaction volumes, each containing 12.5 µl Taq DNA polymerase 2X Master Mix (Ampliqon, Denmark), 1 µl of each oligonucleotide primer, 9.5 µl nuclease-free water, and 1 µl DNA template. PCR products were analyzed by electrophoresis on 2% agarose gel and finally visualized with a gel documentation system (UVP, BioDoc-It Imaging System, USA). Positive PCR products were purified and sequenced by Kawsar Biotech Company (Tehran, Iran). The nucleotides and deduced protein sequence alignment were also performed online using the basic local alignment search tool (BLAST) program of the National Center for Biotechnology Information.

## Results

In this project, 169 *P*. *aeruginosa* isolates were collected from clinical specimens including urine (70 isolates; 41.4%), ETT secretions (51 isolates; 30.2%), blood (19 isolates; 11.2%), wound (18 isolates; 10.6%), throat swab (4 isolates, 2.4%), eye (3 isolates; 1.8%), ureter secretions (2 isolates; 1.2%), ear secretions (1 isolate; 0.6%), and catheter (1 isolate; 0.6%). Eighty-eight (52.1%) isolates were from male and 81 (47.9%) from female patients.

Antimicrobial susceptibility testing demonstrated that 67 isolates were not susceptible to at least one of the carbapenems including imipenem, meropenem, and doripenem, which were therefore selected to be tested by mCIM and CDT. The MIC of imipenem was determined for the 54 imipenem-resistant isolates. The MIC values of imipenem for 53 isolates were ≥32 µg/ml and for one isolate was 8 μg/ml. Out of the above-mentioned 67 isolates, 40 were mCIM positive. Therefore, in the present study, 40 (23.7%) of 169 isolates were carbapenemase producers. Out of 40 carbapenemase-producing isolates, 26 were MBL-positive using CDT with imipenem (CDT-IMI). Thus, based on the phenotypic test, 26 (15.4%) of 169 isolates were MBL producers. The results of CDT-IMI with 930 µg EDTA and CDT-IMI with 750 µg EDTA were the same (Table 2). 

Also, CDT with meropenem (CDT-MEM) was performed in this project. Out of 26 MBL-producing isolates, 5 were positive using CDT-MEM with 930 µg EDTA and 3 were positive using CDT-MEM with 750 µg EDTA (Table 2).

It should be noted that 930 µg and 750 µg EDTA did not show an antibacterial effect around the blank disks.

All 169 isolates were tested by PCR for the detection of MBL genes. The presence of *bla*_IMP_, *bla*_NDM_, and *bla*_VIM_ genes was confirmed via sequencing the PCR products and checking them using the BLAST program of the National Center for Biotechnology Information. Out of 26 isolates that were identified as MBL producers by the phenotypic method, 25 isolates carried the genes studied in this project. *bla*_IMP_, *bla*_NDM_, and *bla*_VIM_ genes were found in 18, 8, and 1 of the MBL-producing isolates, respectively. Coexistence of *bla*_IMP_ and *bla*_NDM_ was observed in 2 MBL-producing isolates. *bla*_NDM_ was detected in 7 carbapenemase-producing isolates that were MBL negative by phenotypic test (CDT negative). *bla*_NDM_ was also detected in 3 isolates that were carbapenemase negative and MBL negative by phenotypic methods (mCIM negative and CDT negative). In addition, *bla*_NDM_ was found in 5 isolates that were susceptible to imipenem, meropenem, and doripenem (Table 3). Among all 169 *P*. *aeruginosa* isolates, 23 (13.6%) harbored *bla*_NDM_, 18 (10.6%) carried *bla*_IMP_, and 1 (0.6%) carried *bla*_VIM_ gene. *bla*_SPM_, *bla*_GIM_, *bla*_AIM_, and *bla*_SIM_ were not found in the present study.

**Table 1:** Primers used in this study for the identification of Pseudomonas aeruginosa and detection of metallo-β-lactamase genes

Primer	Sequence (5′-3′)	Target gene	Product size (bp)	Reference
PA-SS-F	GGGGGATCTTCGGACCTCA	16S rDNA^α^	956	
PA-SS-R	TCCTTAGAGTGCCCACCCG			
VIM-F	GATGGTGTTTGGTCGCATA	*bla*_VIM_	390	
VIM-R	CGAATGCGCAGCACCAG			
IMP-F	GGAATAGAGTGGCTTAAYTCTC	*bla*_IMP_	233	
IMP-R	GGTTTAAYAAAACAACCACC			
NDM-F	GGTTTGGCGATCTGGTTTTC	*bla*_NDM_	621	
NDM-R	CGGAATGGCTCATCACGATC			
SPM-F	AAAATCTGGGTACGCAAACG	*bla*_SPM_	271	
SPM-R	ACATTATCCGCTGGAACAGG			
GIM-F	TCGACACACCTTGGTCTGAA	*bla*_GIM_	477	
GIM-R	AACTTCCAACTTTGCCATGC			
AIM-F	CTGAAGGTGTACGGAAACAC	*bla*_AIM_	322	
AIM-R	GTTCGGCCACCTCGAATTG			
SIM-F	TACAAGGGATTCGGCATCG	*bla*_SIM_	571	
SIM-R	TAATGGCCTGTTCCCATGTG			

^α ^16S rDNA of *Pseudomonas aeruginosa*

**Table 2:** Results of mCIM and CDT in 67 *Pseudomonas aeruginosa* isolates that were intermediate or resistant to one or more carbapenems

No. Isolates	mCIM	CDT-IMI 930 µg EDTA	CDT-IMI 750 µg EDTA	CDT-MEM 930 µg EDTA	CDT-MEM 750 µg EDTA
21	+	+	+	–	–
3	+	+	+	+	+
2	+	+	+	+	–
14	+	–	–	–	–
27	–	–	–	–	–

mCIM: modified carbapenem inactivation method; CDT: combination disk test; CDT- IMI: CDT with imipenem; CDT-MEM: CDT with meropenem

**Table 3:** Frequency of MBL genes among *Pseudomonas aeruginosa* isolates

Gene	mCIM (+), CDT (+) No. Isolates (%)	mCIM (+), CDT (–) No. Isolates (%)	mCIM (–), CDT (–) No. Isolates (%)	NT No. Isolates (%)	Total isolates (%)
*bla*_IMP_	16 (9.5)	0 (0)	0 (0)	0 (0)	16 (9.5)
*bla*_NDM_	6 (3.5)	7 (4.1)	3 (1.8)	5 (2.9)	21 (12.4)
*bla*_IMP, _ *bla*_NDM_	2 (1.2)	0 (0)	0 (0)	0 (0)	2 (1.2)
*bla*_VIM_	1 (0.6)	0 (0)	0 (0)	0 (0)	1 (0.6)

MBL: metallo-β-lactamase; mCIM: modified carbapenem inactivation method; CDT: combination disk test; mCIM (+): mCIM positive; mCIM (–): mCIM negative; CDT (+): CDT positive; CDT (–): CDT negative; NT: isolates that were susceptible to imipenem, meropenem, and doripenem, thus, were not tested by mCIM and CDT

## Discussion

The emergence and spreading of MBL-producing *P*. *aeruginosa* is a universal concern threatening not only immunosuppressed patients but also healthy members of the community. MBL-producing *P*. *aeruginosa* is an important organism due to its antibiotic resistance characteristics as well as its pathogenicity. It carries various antimicrobial resistance genes and is able to transfer these to other strains. Detection of MBL-producing isolates and studies on the frequency of these isolates are recommended in order to develop strategies that control and limit the transmission of MBL-producing *P*. *aeruginosa* (3).

In this study, CDT-IMI and CDT-MEM were used for phenotypic detection of MBL production; both methods were performed using 930 µg EDTA and 750 µg EDTA. The results of the CDT-IMI with 930 µg EDTA and CDT-IMI with 750 µg EDTA were the same, so that 26 MBL-producing isolates were detected. However, CDT-MEM with 930 µg EDTA and with 750 µg EDTA detected 5 and 3 isolates of 26 MBL-positive isolates, respectively. Of the 26 isolates identified by the phenotypic method as MBL producers, 25 isolates harbored MBL genes. Therefore, in our study, the results of CDT-IMI were much better than the results of CDT-MEM. Also, in the study performed by Heinrichs *et al*., CDT with imipenem showed the best results for the detection of MBL-producing *P*. *aeruginosa* (12). In contrast to our results, in the study conducted by Pitout and colleagues, the results of CDT with meropenem were better than the results of CDT with imipenem (5).

As mentioned above, of the 26 isolates identified as MBL producers by phenotypic methods, MBL genes were detected in 25 isolates but not in one. This isolate probably harbored a rare MBL gene such as *bla*_DIM_ (14, 15) that has not been investigated in our study.

In our work, out of 40 carbapenemase-producing *P*. *aeruginosa*, 26 isolates were MBL producers by phenotypic test (CDT positive), whereas 14 isolates were CDT negative. It is noteworthy that *the bla*_NDM_ gene was detected in 7 of these 14 isolates by PCR. Thus, it is probable that these 7 isolates were also MBL producers, but CDT was not able to detect MBL (false negative results).

Based on the results of the phenotypic test, out of 67 isolates that were not susceptible to one or more carbapenems, 26 (38.8%) were MBL producers. The frequency of MBL producers among all 169 isolates using CDT was 15.4% (26/169). In the study performed by Bagheri Bejestani *et al*. in Tehran, Iran, 3.3% of *P*. *aeruginosa* isolates were MBL producers based on CDT results, which was a lower frequency than the frequency obtained from our study (16). In a study conducted by Mirbagheri and colleagues in Mashhad, northeast of Iran, according to CDT results, 88.8% of imipenem-resistant isolates were MBL producers (17). In another study in Ahvaz, southwest of Iran, 90% of imipenem-resistant isolates were phenotypically MBL producers; these researchers also used CDT (1). Therefore, the frequency of MBL-producing isolates in these two studies was higher than the frequency in our study. In an investigation in India carried out by Arunagiri *et al*., 70.1% of multidrug-resistant *P*. *aeruginosa* were MBL producers based on the CDT results (18). In a study in Canada on 241 isolates that were not susceptible to imipenem, 110 (46%) isolates were detected as MBL positive by CDT (5). In Belgium, Heinrichs and colleagues studied 162 multidrug-resistant *P*. *aeruginosa* that were not susceptible to imipenem; 52 (32%) isolates were MBL positive in their investigation. Heinrichs *et al*. used CDT and disk synergy test (DST), but CDT with imipenem showed the best results (12). The reasons for these different results are difficult to explain but may be due to differences in the distribution of MBL genes across various geographical areas, studied isolates, and methods for the phenotypic detection of MBL producers.

In this study, the MIC of imipenem was determined for the 54 imipenem-resistant isolates. The MIC values of imipenem for 53 isolates were ≥32 µg/ml, which indicates a high-level resistance of these 53 isolates to imipenem. Imipenem showed a MIC ≥32 µg/ml for all 26 isolates that were MBL positive by the phenotypic method. In addition, the MIC values of imipenem were ≥32 µg/ml for 9 imipenem-resistant isolates that were MBL negative by phenotypic method but harbored the *bla*_NDM_ gene.

In this study, *bla*_IMP_, *bla*_NDM_, and *bla*_VIM_ genes were detected in 18 (69.2%), 8 (30.8%), and 1 (3.8%) of the MBL-producing isolates, respectively. Among all 169 *P*. *aeruginosa* isolates, 23 (13.6%) harbored *bla*_NDM_, 18 (10.6%) carried *bla*_IMP_, and 1 (0.6%) carried the *bla*_VIM_ gene. *bla*_SPM_, *bla*_GIM_, *bla*_AIM_, and *bla*_SIM_ were not found in our work. In a study in Ahvaz, Iran, *bla*_VIM-2_ and *bla*_IMP-1_ were detected in 1.6% and 55% of imipenem-resistant isolates, respectively, but, no *bla*_SPM-1_ gene was found in the isolates (1). In a study done by Haghi and colleagues in Zanjan, northwest of Iran, the frequency of *bla*_IMP_, *bla*_VIM_, *bla*_SPM_, and *bla*_SIM_ among the MBL-producing isolates was 80%, 17.1%, 57.1%, and 14.1%, respectively. Therefore in contrast to our results, *bla*_SPM_ and *bla*_SIM_ were detected in their study. However, similar to our results, no *bla*_GIM_ harboring isolate was detected. Also, these authors reported that 2 isolates contained *bla*_NDM-1_, *bla*_IMP_, and *bla*_SPM_ (19). Coexistence of *bla*_IMP_ and *bla*_NDM_ was also observed in 2 (7.7%) MBL-producing isolates in our work.

In an investigation conducted by Bagheri Bejestani *et al*. in Tehran, Iran, 3.3% of isolates carried the* IMP* gene (16) which was lower in frequency compared with our study. In the present study, no *bla*_SPM_ gene was identified in any of the isolates which was in accordance with the study performed by Yousefi *et al*. in Tabriz, northwest Iran (20). In the study conducted in Brazil, among the MBL-producing isolates, 55.6% were positive for *bla*_SPM-1_ (21).

In an investigation in Iraq, *bla*_NDM_ was detected in 4 of 22 *P*. *aeruginosa* isolates (4). In the study done in India, 17.3% of carbapenem-resistant *P*. *aeruginosa* isolates carried *bla*_NDM-1_ (22). In our work, among all 169 *P*. *aeruginosa* isolates, *bla*_NDM_ was the most frequently detected MBL gene which emphasizes the need for surveillance and precautions.

It should be noted that in our study *bla*_NDM_ was identified in 5 isolates that were susceptible to imipenem, meropenem, and doripenem (Table 3). The reason for susceptibility of these isolates to carbapenems despite the presence of the *bla*_NDM_ is unclear. These isolates may be silent reservoirs of the *bla*_NDM_. A similar report on *the bla*_SPM_ gene has been published by Pellegrino and colleagues. These authors reported a carbapenem-susceptible *P*. *aeruginosa* strain that carried *bla*_SPM_; they stated that such isolates may be silent reservoirs of the *bla*_SPM_ gene (23).

## Conclusion

*bla*_NDM_, *bla*_IMP_, and *bla*_VIM_ genes were detected in this study which could be a warning sign about the prevalence of these genes among *P*.* aeruginosa* clinical isolates in our region. Proper monitoring and detection of MBL-producing isolates are the essential steps to prevent the spread of these isolates. 

## Authors’ Contributions

FY, BN, and ST designed the experiments; MS, FY, and ST performed experiments and collected data; MS, FY, BN, and ST discussed the results and strategy; FY and ST Supervised, directed, and managed the study; MS, FY, BN, and ST approved the final version.

## Conflicts of Interest

The authors declare that there are no conflicts of interest.
